# A Case Study of the New York City 2012-2013 Influenza Season With Daily Geocoded Twitter Data From Temporal and Spatiotemporal Perspectives

**DOI:** 10.2196/jmir.3416

**Published:** 2014-10-20

**Authors:** Ruchit Nagar, Qingyu Yuan, Clark C Freifeld, Mauricio Santillana, Aaron Nojima, Rumi Chunara, John S Brownstein

**Affiliations:** ^1^Children's Hospital Informatics ProgramBoston Children's HospitalBoston, MAUnited States; ^2^Yale UniversityNew Haven, CTUnited States; ^3^Management SchoolUniversity of Chinese Academy of SciencesBeijingChina; ^4^Boston UniversityBiomedical Engineering DepartmentBoston, MAUnited States; ^5^Harvard UniversitySchool of Engineering and Applied SciencesCambridge, MAUnited States; ^6^Harvard UniversitySchool of Public HealthBoston, MAUnited States; ^7^Massachusetts Institute of TechnologyCambridge, MAUnited States; ^8^Department of PediatricsHarvard Medical SchoolBoston, MAUnited States

**Keywords:** influenza, Twitter, New York City, spatiotemporal, Google Flu Trends, infodemiology, mHealth, social media, natural language processing, medical informatics

## Abstract

**Background:**

Twitter has shown some usefulness in predicting influenza cases on a weekly basis in multiple countries and on different geographic scales. Recently, Broniatowski and colleagues suggested Twitter’s relevance at the city-level for New York City. Here, we look to dive deeper into the case of New York City by analyzing daily Twitter data from temporal and spatiotemporal perspectives. Also, through manual coding of all tweets, we look to gain qualitative insights that can help direct future automated searches.

**Objective:**

The intent of the study was first to validate the temporal predictive strength of daily Twitter data for influenza-like illness emergency department (ILI-ED) visits during the New York City 2012-2013 influenza season against other available and established datasets (Google search query, or GSQ), and second, to examine the spatial distribution and the spread of geocoded tweets as proxies for potential cases.

**Methods:**

From the Twitter Streaming API, 2972 tweets were collected in the New York City region matching the keywords “flu”, “influenza”, “gripe”, and “high fever”. The tweets were categorized according to the scheme developed by Lamb et al. A new fourth category was added as an evaluator guess for the probability of the subject(s) being sick to account for strength of confidence in the validity of the statement. Temporal correlations were made for tweets against daily ILI-ED visits and daily GSQ volume. The best models were used for linear regression for forecasting ILI visits. A weighted, retrospective Poisson model with SaTScan software (n=1484), and vector map were used for spatiotemporal analysis.

**Results:**

Infection-related tweets (*R*=.763) correlated better than GSQ time series (*R*=.683) for the same keywords and had a lower mean average percent error (8.4 vs 11.8) for ILI-ED visit prediction in January, the most volatile month of flu. SaTScan identified primary outbreak cluster of high-probability infection tweets with a 2.74 relative risk ratio compared to medium-probability infection tweets at *P*=.001 in Northern Brooklyn, in a radius that includes Barclay’s Center and the Atlantic Avenue Terminal.

**Conclusions:**

While others have looked at weekly regional tweets, this study is the first to stress test Twitter for daily city-level data for New York City. Extraction of personal testimonies of infection-related tweets suggests Twitter’s strength both qualitatively and quantitatively for ILI-ED prediction compared to alternative daily datasets mixed with awareness-based data such as GSQ. Additionally, granular Twitter data provide important spatiotemporal insights. A tweet vector-map may be useful for visualization of city-level spread when local gold standard data are otherwise unavailable.

## Introduction

Seasonal influenza epidemics result in an estimated three to five million cases of severe illness and 250,000 to 500,000 deaths worldwide each year [1]. In order to better control seasonal influenza epidemics and the next pandemics, researchers have proposed several “infodemiological” approaches [2,3] to achieve near real-time surveillance with Internet data, such as Google search query (GSQ) [4-8] and textual Twitter data [9-15].

The idea of using GSQ data volume for detecting outbreaks was first introduced in 2006 by Eysenbach et al [4], then refined by Ginsburg et al with the Google Flu Trends project [5]. Until recently, Google Flu Trends has matched weekly influenza-like illness (ILI) incidence well, but the recent 2012-2013 flu season in the United States was overestimated by 200% [16]. Though providing impetus for increased vigilance during one of the worst flu seasons to date, this case demonstrates that Google Flu Trends alone has limitations for influenza prediction, and motivates search for improved models [17,18]. New models can look for refined algorithms but may also seek out independent datasets to improve predictive abilities. With personal, textual data from potentially sick tweeters, Twitter offers a rich alternative dataset. Twitter epidemic surveillance was first introduced by Ritterman et al (2009) [9], who showed that monitoring tweets can improve the accuracy of market forecasting models by providing early warnings of external events like the H1N1 outbreak. Chew and Eysenbach subsequently evaluated the content and sources of the Twitter response to the swine flu outbreak in 2009, seeing potential for public health insights [19]. Recent papers from Culotta [20,21], Signorini [22], Vadileios [10], Kim [12], Santos [23], and Achrekar [15] et al have shown the usefulness of Twitter data to retrospectively predict national and regional influenza cases on a weekly basis. Broniatowski and colleagues have recently considered Twitter’s temporal predictive strength for cases at the municipal level in New York City, with a prospective approach [24].

The promise of Twitter as a mechanism for timely signal detection has spawned efforts to build tools for surveillance, including MappyHealth [25], germTracker [26], Observatorio da Dengue [27], Infovigil [28], and SickWeather [29]. But studies of Twitter’s temporal predictive capabilities have not adequately addressed how the information can be brought to a concrete, local scale. In this paper, we look to dive deeper into the case of New York City (NYC) by analyzing daily Twitter data from temporal and spatiotemporal models. We look for novel insights by being the first to consider daily data (Twitter and GSQ) for influenza case predictions and by using geolocated tweets to estimate the probability of flu transmission within the city.

## Methods

### Data Sources

Official influenza-like illness emergency department visit (ILI-ED) counts were obtained from the Weekly Influenza Surveillance Reports released by the municipal government of the city of New York [30]. These reports were compiled into a daily breakdown. The data for the ILI cases was extracted from the graphs using optical plot reading software (WebPlotDigitizer [31]) validated by pixel counting for each daily value. Data for total counts for NYC and counts for each borough were determined by this method. This method was employed because data were otherwise unavailable from the NYC Department of Health.

### Google Trends Search Query Data

Google Trends provides a time series index of the volume of queries users enter into Google in a given geographic area. The query index is based on query share: the total query volume for the search term in question within a particular geographic region divided by the total number of queries in that region during the time period being examined. The maximum query is normalized to 100 and the query share at the initial date being examined is normalized to be zero [7]. This query share also varies with time. In our model, the search queries used were “flu”, “gripe”, “influenza”, and “high fever”. According to our survey, these queries gave the highest signal. More importantly, these exact keywords were also used for tweet collection as a basis for comparison. Google provides weekly data for state and (limited) city searches. Daily data had to be extracted by querying the search volume over overlapping time periods and proportionally adjusting the query index along the time series. The Trends data were all downloaded within a single day (May 11, 2013), as Google varies the signal display with time. It is important to note that these data are distinct from the weekly Google Flu Trends (GFT) data, which does not provide a daily breakdown and was not used in this study. That being said, GSQ could act as a potential proxy for GFT. When comparing the 7-day GSQ volume totals with GFT weekly volume hits, their correlation coefficient was .78 between September 23, 2012 and May 5, 2013.

### Twitter Data

Using the Twitter Streaming API, geocoded tweets were collected from October 15, 2012 to May 10, 2013; unlike the method suggested by Broniatowski and colleagues, our approach did not involve normalizing the flu-related tweets against the weekly or daily total Tweet count. This was not done because we saw variation in the baseline number of tweets per day and because the daily volume of tweets was low (ranging from 0 to 120 tweets at peak season).

These tweets were selected based on the geographical bounding box from (40.44, −74.93) to (41.12, −72.63). This window was chosen to account for people commuting into New York City from New Jersey and Long Island. The assumption was that the signal position of tweets would not change significantly by moving the bounding box further out from the city; however, the larger area would allow for greater tweet volume for analysis. To determine if the tweet was inside the frame of interest, the tweet latitude and longitude were first checked. If the tweet location was missing, the profile latitude and longitude was used. Users could also define a text-based location for their profile, but these tweets could not be reliably determined to be inside the bounding box, and were therefore excluded. Dredze et al have developed “Carmen”, a system to geolocate tweets by cross-referencing location keywords to a database, but there remains difficulty in comparing tweets with a GPS stamp to those which are geolocated to a broader region [32].

Keyword filters were then applied to our collection of tweets: “flu”, “gripe”, “influenza”, and “high fever” were case-insensitive, word-bounded inclusion strings. “Avian”, “stomach”, and “bird” were exclusion strings. Although the purpose of this study was not to optimize keyword selection, preliminary studies were conducted to see the effect on the signal to noise ratio when adding inclusion words for other ILI symptoms and medications. Ginsberg and colleagues methodically constructed a set of 45 significant keywords for Google Flu Trends using a linear regression model [5]. Similar methods have been used by Kim et al for Hangeul Twitter [12]. Optimal keywords, however, vary across time and geographical region, so our approach focused on obtaining a signal that was both strong and specific and was not concerned with the decreasing marginal value of the next best keyword. Our search was broad enough to cover the top 11 ILI-related search query topics that grouped the Google Flu Trends 45 keywords.

After collecting the filtered tweets, all 2972 tweets were manually curated. Duplicate tweets from the same user were first removed from the dataset. We then created categories for Twitter classification using the models established by Lamb et al [33]: Relevant (R) vs Irrelevant (Ir), Awareness (A) vs Infection (I), and Self (S) vs Other (O). Additionally, a fourth option (not included by Lamb et al) was added to mark High (H) vs Medium (M) vs Low (L)—a guess for the probability of the subject(s) mentioned being sick. This category could help differentiate a sarcastic tweet from a serious one, by assigning a rank to the veracity of the subject being sick. A user-based guess for the sickness of the subject has also been employed by germTracker and CrowdBreaks to leverage human-based classification of tweets [34].

All tweets were labeled after being dissociated with their dates to prevent bias of an anticipated regular flu season on categorization. The categories were grouped into 12 four-letter codes and an additional code for irrelevant tweets. In general, Relevant-Infection-Self-High/ Relevant-Infection-Self-Medium (RISH/RISM) and Relevant-Infection-Other-High/ Relevant-Infection-Other-Medium (RIOH/RIOM) constituted tweets for people who had the flu or flu-like symptoms; Relevant-Infection-Self-Low/ Relevant-Infection-Other-Low (RISL/RIOL) grouped tweets for people who were recovering from illness; Relevant-Awareness-Self-High/ Relevant-Awareness-Self-Medium (RASH/RASM) generally grouped tweets of people with negative reactions to flu shots; Relevant-Awareness-Self-Low (RASL) grouped tweets for flu shots and successful vaccinations; Relevant-Awareness-Other-High / Relevant-Awareness-Other-Medium (RAOH/RAOM) referred to news media alerts of different severity; and Relevant-Awareness-Other-Low (RAOL) grouped tweets focused on public health awareness and therapies. Examples of this scheme can be seen in Multimedia Appendix 1.

### Ethics

The tweets used in this study were publicly distributed. Consent for these tweets to be read comes from users signing the Twitter Terms and Agreement and agreeing to public privacy settings. This project was exempted from IRB approval since it did not meet the criteria for human subjects’ research. Nevertheless, for the purpose of this study, tweeters’ user IDs were not collected so each tweet entity remained anonymized. The subsequent analyses were dependent on the tweet content, tweet frequency (aggregate), and tweet location.

### Predictive Models

#### Temporal

First, Pearson correlation values were constructed between each of the Twitter category time series and the GSQ time series to the ILI-ED time series for New York City. Next, to forecast the ILI data, AR (auto-regressive) models were used. ILI was the dependent variable, while GSQ and the strongest category Twitter data were the independent variables separately in different models. Each model was tested for 7 weeks between January 6, 2013 and February 23, 2013 to compare the predictive abilities in ILI visits during the volatile peak of the flu season.

#### Spatiotemporal (Retrospective)

Since the selected tweets are geocoded and include a date stamp, they are also fact space-time data. Using SaTScan software [35], we were able to perform retrospective geographical surveillance of the 2012-2013 flu season to detect statistically significant space-time disease clusters. The space-time test statistic is defined by a cylindrical window with circular base. The circular base represents the spatial scan, with the base varying from zero to a size that captures 50% of the population risk for a given tweet. The set of circular bases scan the map for potential clusters. Similarly, the height of the cylinder represents the temporal progression of the map varying from 0 to 50% of the total time period. The cylinder is moved both laterally in space and vertically in time (aggregated in weekly steps) to identify possible clusters for the entire study region to generate the test statistic.

A total of 1484 total RISM and RISH tweets, restricted to New York City proper, were used for the spatiotemporal analysis between October 15, 2012 and May 10, 2013. RISH tweets were treated as the potential flu cases, while RISM tweets were treated as control cases. These tweets were also weighted based on their seasonal time-series correlation value to gold standard ILI visits (RISH=.689, RISM=.655) to better reflect their relative association with real flu cases. After assigning weights, a Poisson probability model was used to search for high clusters with an excess of RISH tweets (compared to control RISM tweets), for each cylinder in the scan window. Both primary and secondary clusters were identified based on a likelihood ratio test statistic using the methods described by Jung and Kulldroff [36].

#### Spatiotemporal (Prospective)

SaTScan provides prospective analysis functionality to predict regions with higher risk ratio. We looked at representing these results in an alternative fashion, in a manner accessible from a Web application. The approach was to construct a vector-map for the larger NYC region. The idea was to use an analogy of a weather map where winds move from areas of high to low pressure. Pressure was modeled by density of potentially sick tweeters and winds represented the direction of change locally in that density. Specifically, for each 0.1 x 0.1 decimal degree grid, the weekly change in percent of flu-related tweets was calculated. A vector was drawn to point in the direction of the neighboring cells with the highest positive percentage increase in flu-related tweets, away from cells with a percentage decrease. Red corresponds with higher percent increase. The underlying assumption of the model was that infection spreads locally between neighboring regions. While it may be argued that disease transmission is not spatially continuous, this model demonstrates one of many possible representations of flu dynamics at the city-level scale (hence the grid dimensions), and how a real-time public health tool may be displayed. Sadilek et al have suggested the importance of colocation with other sick tweeters (friends or otherwise) in their individual-based model of spatiotemporal prediction. Therefore, understanding changes in sick tweeter colocation is importantly indicated by our wind-map. The macro-level validation of our model will come only with the availability of more gold-standard, spatiotemporal data.

Further discussion of spatial models of temporal windows and their relation to spatial predictor covariates (age, ethnicity, population density, distance to school and subway, distance from home, distance from vaccination sites) can be found in Figures 8 and 9 in Multimedia Appendix 2.

## Results

### Temporal

The time series of tweet counts was calculated to first assess the quality of the data. We classified over 90% of the tweets collected as relevant. Most tweets were about the self (S), about being infected (I) or having ILI symptoms, and were ranked with high probability (H) for the subject being sick, as seen in Table 1. These Pearson correlation values correspond to tweets from October 15, 2012 to May 10, 2013.

**Figure 1:**
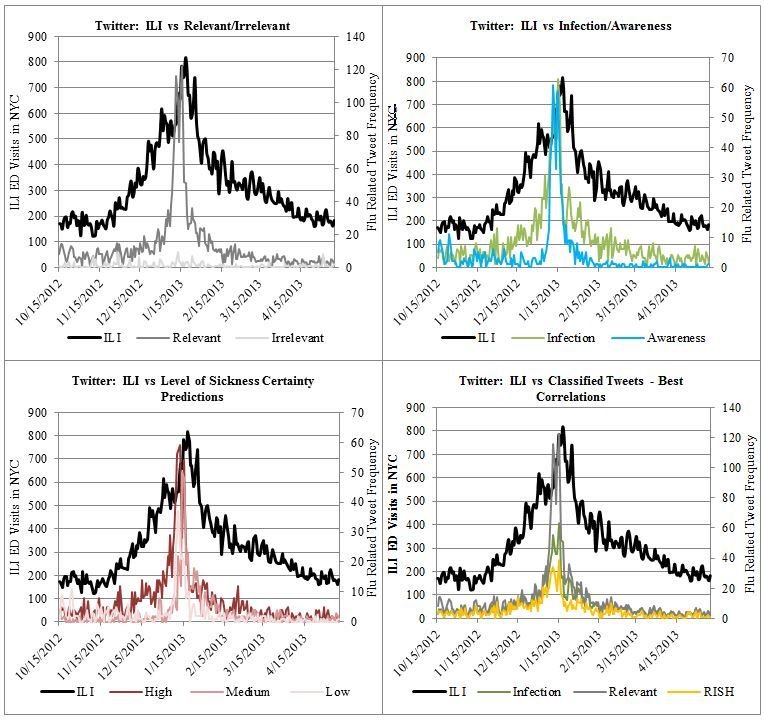
Time series comparisons between Tweet categories and ILI-ED visits.

Each time series, including the GSQ time series, was then compared to the ILI data and ranked (as seen in Table 1). Infection, RISH, and Relevant groups and subgroup had higher Pearson correlations with the gold-standard ILI data between October 15, 2012 and May 10, 2013. Figure 1 illustrates aforementioned relationships with the ILI data.

**Figure 2:**
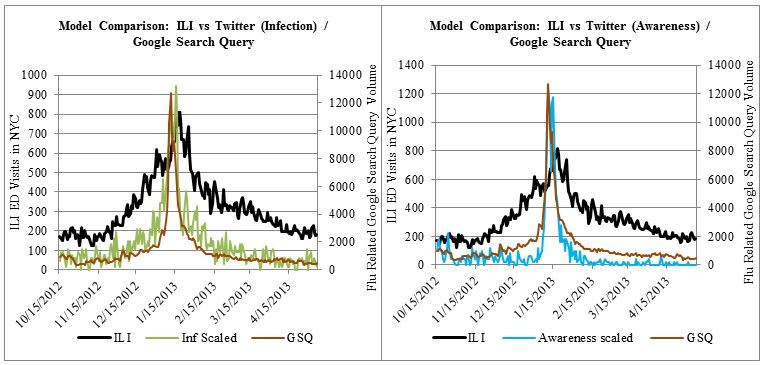
Comparison of Infection tweets and Awareness-based data.

Figure 2 illustrates the comparison between GSQ and Twitter data, comparing both to ILI. Note the Pearson correlation between GSQ and Twitter “Awareness” time series is .934. Both have a characteristic spike on January 10, 2013, the day the city of Boston declared a public health emergency for influenza.

**Table 1:** Quality of the classified tweets and search query data.

Tweet group^a^		Percentage of tweets	Time series	Pearson correlation
Relevant		0.907	Infection	.763
Self		0.689	RISH	.689
Infection		0.628	Relevant	.687
High		0.497	GSQ	.683
Awareness		0.279	Self	.677
Medium		0.223	Medium	.668
Other		0.219	Other	.666
Low		0.188	High	.665
Irrelevant		0.082	RISM	.655
			RIOH	.616
**Sub Group**			RAOM	.587
	RISH	0.399	Awareness	.549
	RASL	0.107	RASM	.545
	RISM	0.100	RISL	.542
	RAOM	0.058	RIOM	.511
	RIOH	0.054	Low	.451
	RISL	0.041	RAOH	.411
	RAOH	0.040	RASL	.351
	RASM	0.037	RASH	.322
	RAOL	0.032	RAOL	.277
	RIOM	0.027	RIOL	.254
	RIOL	0.007	Irrelevant	.213
	RASH	0.005		

^a^Relevant (R), Awareness (A), Infection (I), Self (S), Other (O), High (H), Medium (M), Low (L).

### Linear Regression Models

In order to compare the Twitter, GSQ, and ILI time series datasets, we first used an Augmented Dickey-Fuller (ADF) test with EVIEWS-7 statistical package to confirm the presence of a unit root. Each dataset was then adjusted to meet the stationarity test. The datasets were not stationary at the 1% significance level until after first order differencing was performed (Tables 2 and 3).

An Englemen Granger co-integration test was then performed on the time-lagged datasets. Establishing co-integration and stationarity allowed for determination of consistent estimators in our regression model. One weakness was present in our approach: seasonality was not tested because the available data only comprised one season. A discussion of the weekday effect and why weekly cyclic terms were not included in the model can be found in Multimedia Appendix 2.

The first AR model incorporates infection time series data and 14-day time-lagged ILI data for the first week. The time lag reflects the real delay in publicly reporting updated ILI-ED visit counts. The model and results are shown in Table 4. Note mu and epsilon are terms for error and drift respectively in the subsequent models.

Model 1: ILI=*m*_1_ILI(−14)+*m*_2_infection(−1)+*m*_3_ *μ*(−1)+*ε*

The results from the model above show that the time-lagged ILI data were not significant for the prediction. This persuaded us to modify the model to incorporate only Infection tweets time series data. Across all 7 weeks, the best model for the Infection tweets time series had the following form:

Model 2: ILI=*α*_1_infection(−2)+*α*_2_ *μ*(−1)+*α*_3_ *μ*(−2)+*ε*

We repeated the same process for GSQ data, constructing a model to incorporate both it and ILI time-lagged data for the first week. The results are listed in Table 5.

Model 3: ILI=*β*_1_ILI(−14)+*β*_2_gsq(−3)+*β*_3_ *μ*(−1)+*β*_4_ *μ*(−2)+*ε*

Again we found the time-lagged ILI data to be not significant and eliminated it from the subsequent GSQ models. For the Google search query data, the following models were constructed to optimize its predictive scores by adjusting the time series lag.

Model 4: ILI=*γ*_1_gsq(−3)+*γ*_2_ *μ*(−1)+ *γ*_3_ *μ*(−2)+*ε* (first and second weeks)

Model 5: ILI=*γ*_1_gsq(−4)+*γ*_2_ *μ*(−1)+ *γ*_3_ *μ*(−2)+*ε* (third to seventh weeks)

The two GSQ and the Twitter Infection models were then compared by their mean absolute percent error or MAPE (see Table 6). The data suggest lower MAPE scores for Twitter for the first 4 weeks in January (4.7, 6.9, 11.8, and 10.4) compared to GSQ data (5.5, 15.8, 12.4, and 11.3).

**Figure 3:**
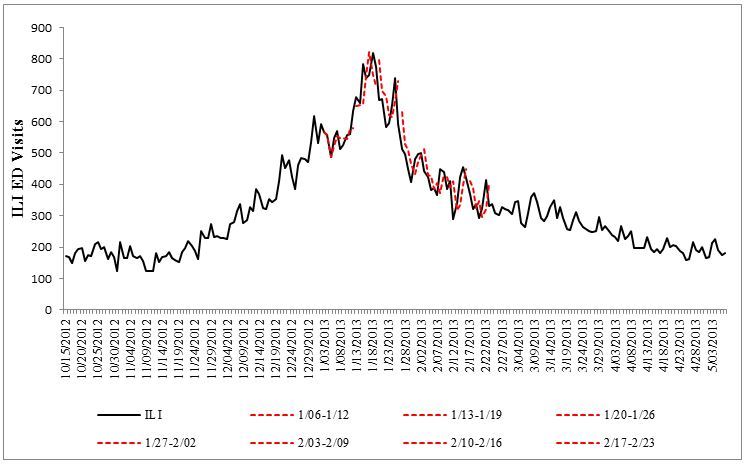
Predicted ILI-ED visits in red using the Infection tweets model (Model 2).

The average MAPE for temporal predictions using the Infection tweet time series was 8.4. Figure 3 demonstrates the ILI predictions using the Infection tweet time series (model 2) for the month of January.

**Table 2:** Augmented Dickey-Fuller (ADF) test of ILI^a^, Twitter, and Google search query data.

		ILI		Twitter infection		Google search query	
		*t* statistic^b^	probability	*t* statistic^b^	probability	*t* statistic^b^	probability
ADF test		−1.902	0.331	−2.569	0.101	−2.844	0.054
**Test critical values**							
	1% level	−3.462		−3.463		−3.463	
	5% level	−2.876		−2.876		−2.876	
	10% level	−2.574		−2.574		−2.574	
		Non-stationary		Non-stationary		Non-stationary	

^a^ILI: influenza-like illness

^b^Degrees of freedom=203

**Table 3:** Augmented Dickey-Fuller (ADF) test of ILI^a^, Twitter, and Google search query data with first order lag.

		ΔILI^b^		ΔTwitter infection^b^		ΔGoogle search query^b^	
		*t* statistic^c^	probability	*t* statistic^c^	probability	*t* statistic^c^	probability
ADF test		−12.544	0.000	−19.358	0.000	−6.920	0.000
**Test critical values**							
	1% level	−3.463		−3.463		−3.463	
	5% level	−2.876		−2.876		−2.876	
	10% level	−2.574		−2.574		−2.574	
		Stationary		Stationary		Stationary	

^a^ILI: influenza-like illness

^b^Δ=first order lag

^c^Degrees of freedom=202

**Table 4:** Results of model (1).

Variable	Coefficient	Standard error	*t* statistic^c^	Probability
Infection(−1)	−2.174	1.016	−2.140	0.036
ILI^a^(−14)	0.224	0.142	1.576	0.120
AR^b^(1)	1.007	0.016	61.676	0.000

^a^ILI: influenza-like illness

^b^AR: auto-regressive

^c^Degrees of freedom=188

**Table 5:** Results of model (3).

Variable	Coefficient	Standard error	*t* statistic^d^	Probability
GSQ^a^(−3)	0.069	0.031	2.218	0.030
ILI^b^(−14)	0.212	0.147	1.444	0.154
AR^c^(1)	0.690	0.125	5.515	0.000
AR(2)	0.315	0.127	2.476	0.016

^a^GSQ: Google Trends search query

^b^ILI: influenza-like illness

^c^AR: auto-regressive

^d^Degrees of freedom=188

**Table 6:** MAPE^a^ scores for Infection tweet and GSQ^b^ models for ILI^c^ predictions.

Date	Twitter model		GSQ models	
	Durbin-Watson statistic	MAPE (static)	Durbin-Watson statistic	MAPE (static)
1/06-1/12	2.00	4.7	2.04	5.5
1/13-1/19	2.11	6.9	2.13	15.8
1/20-1/26	2.16	11.8	2.16	12.4
1/27-2/02	2.07	10.4	2.04	11.3
2/03-2/09	2.09	8.2	2.06	7.9
2/10-2/16	2.08	14.8	2.05	15.2
2/17-2/23	2.08	15.3	2.05	14.5

^a^MAPE: mean absolute percent error

^b^GSQ: Google Trends search query

^c^ILI: influenza-like illness

### Spatiotemporal Models

**Figure 4:**
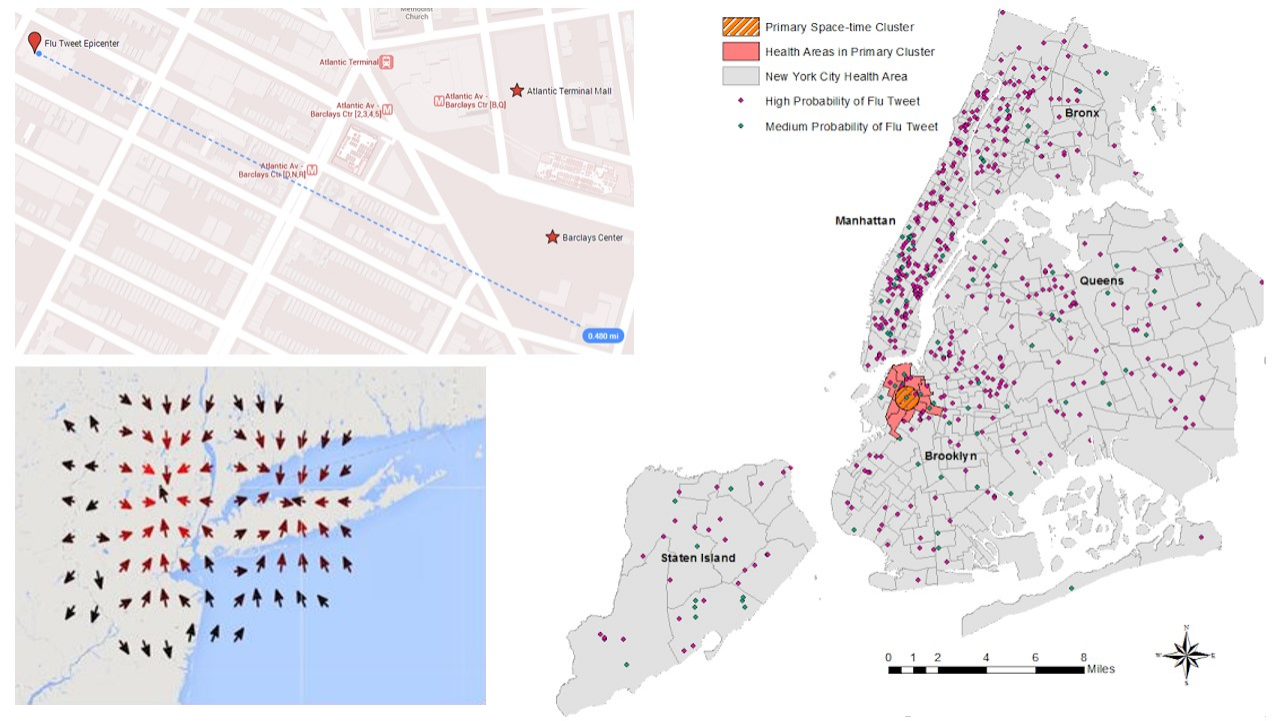
Right: Retrospective primary space-time cluster (p < .001) for high risk of tweeting flu infection-based Content, determined by a Poisson Model with cases as High Probability Flu Tweets and controls as Medium Probability Flu Tweets, aggregated by week, and with content-specific covariate weight in NYC during 10/15/2012-5/10/2013. Top Left: Epicenter located at (40.685, -79.983) with 0.48 mile radius including places of mass gathering such as Barclays Center and Atlantic Avenue Terminal. Bottom Left: Prospective approach to modeling weekly changes in local Infection-tweet spread.

From the retrospective analysis [37], a primary space-time cluster for RISH tweets was found in North Brooklyn [38] with a relative risk of 2.74 (RISH to control RISM) between November 24, 2012 and March 11, 2013 at a significance of *P*<.001. The prospective vector-map was constructed for the week following January 8-15, 2013 and can be seen in Figure 4.

## Discussion

### Principal Results

The principal aim of this study was to evaluate the strength of Twitter data in predicting flu cases on a daily local level in New York City. Our method for selecting this Twitter data with keyword filters and location filters returned a dataset with 90%+ relevant tweets. Daily Infection tweets showed the highest correlation with daily ILI visits (*R*=.763) for the 2012-2013 time period. While we believed that the RISH category most clearly identified personal accounts of sickness, the correlation with ILI was not as strong as using just the Infection tweets umbrella (*R*=.689). This may be due to the fact that Infection aggregates reports from both individuals and others to better reflect total cases of flu across various levels of certainty based on level of symptom progression. Low correlation values for the RISL group (.542) compared to the correlations for RISM and RIOH (.616 and .655), however, still are consistent with the evaluator guess in the classification scheme.

Twitter data (Infection and RISH) importantly outperformed daily GSQ data (*R*=.683) with respect to correlation to daily ILI visits between October 15, 2012 to May 10, 2013. Qualitatively these results are consistent with our expectations. Twitter allows infection-related testimonials to be extracted. These Infection tweets are better predictors of potential cases than Awareness tweets (Figure 1) and GSQ data, neither of which can distinguish people who search due to awareness or from infection. In fact, GSQ resembles Awareness tweets (Figure 2), with a correlation of .934. Both datasets had large increases (about 600%) in volume immediately after the nearby January 10 Boston public health emergency declarations (the New York state of emergency was announced 2 days later and also showed a spike). Similar spikes in public attention on Twitter after media releases have been noted by Gu and colleagues in their analysis of the 2013 H7N9 outbreak in China, with the most pronounced effect in the first 3 days [39]. From a public health standpoint, an awareness reaction to a media response provides information that is either exaggerated, already established, or both. Infection tweets are more relevant because they indicate current probable cases not necessarily accounted for by the hospital network. This is not to entirely discount Awareness tweets. In fact, Awareness-related tweets can still provide important insight on vaccination. The data from Relevant, Awareness, and Self categories suggests people tweeted about flu shots when it was probably too late (see Figure 2 in Multimedia Appendix 2). Although there was some signal from October to December, tweets about the flu shot peaked near peak flu season, at which point those tweeters would have already been exposed to the virus with weakened immunity. It is possible that delayed vaccinations contributed to the increased intensity of the peak season.

When considering temporal forecasting, Infection tweets performed better than the GSQ data. While the predictions were comparable for the off-peak flu season, the difference in MAPE was apparent between January 6 and February 23, 2013, which included the peak and highest volatility of the flu season. The Infection tweet model had an average MAPE of 8.4 compared to GSQ models that had a MAPE of 11.8. Importantly, available and officially released time-lagged ILI data were found to be not statistically significant in predicting real-time ILI cases. This further underscores the need for alternative real-time data sources such as Twitter.

Spatiotemporal analysis also provided valuable insights. In particular, a primary cluster of a high ratio of high-probability sick tweeters to medium-probability infection tweeters was found in northern Brooklyn across a timeframe from November through March. This cluster includes Barclay’s Center and the Atlantic Avenue Terminal—both places of mass gathering and commute and therefore increased probability of infection and/or transmission of influenza. It is not definitive what factors led to an increased propensity to tweet high-confidence infection tweets or by extension what factors led to increased sickness in that cluster. The New York City Department of Health does have daily time series data for each borough (see Figure 3 in Multimedia Appendix 2). The Bronx, Manhattan, Brooklyn, and Queens all tend to follow similar trends in the overall ILI-ED visits as the whole of NYC; each of the four boroughs shares the peak flu visits for the period between January 8 and January 18, 2013. Staten Island has far fewer reports and no discernible peak. Aggregating the Department of Health time series data on the borough level may not immediately reveal knowledge of potential clusters, such as the one identified in Northern Brooklyn by SaTScan using geocoded tweets. Prospective models, like the one in this study (see Figure 4), have yet to be validated due to lack of geographical gold standard data for comparison. We believe that the representation however is useful in demonstrating one possible model of local diffusion.

### Limitations

This study faced several limitations. Classifying the tweets underscored that textual interpretation is a difficult task that requires human interpreters with contextual knowledge of the language and region of interest. The ability to recognize slang, misspellings, Twitter lexicon, inside references, current events, intention, and mood of the tweet sets a high threshold for extracting meaning and sentiment for machine-learning algorithms, experienced researchers, and contracted data classifiers alike. For metropolitan areas that have higher tweet density, multiple languages can come into play. The word “gripe” for example can mean complaint in English but influenza in Portuguese and Spanish; it is also a misspelling of “grippe” in French. Moreover, the tweets were only queried in English and Spanish. With already low tweet volume, capturing other languages such as Italian, Portuguese, Malay, and Tagalog will be needed to refine models moving forward. When it comes to qualitative coding, checking for inter-rater reliability is key as the process is inherently subjective. We are attaching our dataset in Multimedia Appendix 3 to improve the feedback of the classification scheme.

The classification approach used here also was manual and the keyword choice was not optimized through iterative deletion from a large bank of keywords. That being said, the purpose was not to obtain the highest correlative value as optimal keywords vary in time frame and region. While the manual approach used here has advantages of eliminating false positives/negatives that may result from automatic classification (as has been commonly reported [40]), the tradeoff of this approach comes in speed of analysis. Automatic classifiers have been successful and can be trained to include additional search strategies for influenza from this study (see Table 1 in Multimedia Appendix 2) to increase speed and accuracy. These rule-based heuristics supplement a growing base of classifier findings by Paul et al [33] and Nagel et al [41], which show bag-of-words, URL Web addresses, retweet status, emoticons, and syntactical organization of a tweet as indicators of illness.

Finally, limitations were present in the modeling approaches. For temporal modeling, seasonality factors were not considered in the time series analysis. This was due to lack of retrospective ILI and Twitter data access. Since influenza is cyclical, seasonality concerns are of extreme relevance in predicting the weeks of the influenza peak. For spatial models, geocoded tweets are few in volume, presenting a clear limitation to the power of the analysis. While many tweeters in New York City may also be tweeting they are sick (without a geocode), at the moment, it is not possible to verify that they are indeed tweeting from within New York City with the available data. Geocoded tweets are, however, expected to grow in the coming years and with this increase comes the potential for higher statistical power [42]. Moreover, Carmen and other text-mining approaches are being considered to increase the fidelity of non-GPS, location-based data [32,43]. From our survey, New York City had the highest volume of geocoded tweets (from 2-3% of total tweets) [42], so it is not clear if the models used here will hold for other cities where data are less prevalent. It is encouraging that in this case study the number of tweets found statistically significant spatiotemporal clusters and temporal autocorrelations at the *P*=.002 and *P*=.01 levels respectively based on the sample. But even with increased geolocation of tweets, when it comes to tracking disease within cities, two obstacles remain: how to verify tweet content, and how to account for tweeter mobility to treat Infection tweets as footprints rather than static incidents. Interactions with the disease could result from interactions within familiar networks of people or from commuting across vast environments.

### Comparison to Prior Work

This is not the first study to demonstrate the relevance of Twitter in predicting influenza cases. Broniatowski and colleagues’ recent examination of Twitter in New York City can be used as a basis of comparison for the temporal analysis [24]. Their algorithm for Infection tweets found a stronger correlation to ILI visits than the approach here (*R*=.88 vs *R*=.763). Broniatowski et al’s simpler keyword filter algorithm, however, has a lower correlation value with ILI visits (*R*=.72). These comparisons, however, are not *ceteris paribus*. In this study, daily, not weekly data are correlated to see for the first time if daily data have sufficient signal at the municipal level. It is also not clear which keywords are being employed in their algorithms and how to compare the forecasting models used without MAPE scores for a given week’s predicted ILI counts.

From a spatiotemporal basis, it is important to consider how this work relates to the framework proposed by Sadilek et al [44]. The approach here attempts to simply map geographical risk based on density of tweets in a given region. Our models would suggest that colocation with high density RISH tweets would suggest higher risk of contracting the disease. Sadilek and colleagues have included colocation with people in social networks as a facet of their model. Our retrospective and prospective models lacked a covariate to measure this network interaction, and thus leave room for improvement. At the same time, the limited number of publicly available geocoded tweets may suggest that such a framework is difficult to implement at the municipal level for a short time period.

Finally, we see an avenue for improving the classification scheme established by Lamb et al by including an evaluator guess. This factor can account for sarcasm, tone of voice, and confidence of the statement made by the tweeter. This factor was also crucial in establishing a basis for case versus control “sick” tweets for spatiotemporal analysis.

### Conclusions

This study presents several major takeaways. It is the first study to consider daily city-level Twitter data as a means of forecasting real-time ILI emergency department visits in New York City. It also suggests useful models for leveraging the geocoded Twitter data to understand potential hotspots of disease (such as the Barclay’s Center and Atlantic Avenue Terminal) as they move throughout the flu season. This information will be relevant in considering availability and access to vaccination sites and monitoring ongoing vaccination rates. Twitter can also inform public health officials of the local, upcoming burden of disease. Public health officials already use SaTScan with electronic medical record (EMR) data to track anomalous outbreaks of disease in space and time. Now, Twitter can provide weighted potential cases from personal reports to improve these models. When hyperlocal, confirmed data on flu cases is otherwise unavailable, Twitter provides a real-time information data source. This information can be filtered to select for infection-specific testimonials and as a dataset, outperforms awareness-mixed, daily data from Google Trends search query. This data can also be leveraged in unique prospective models to forecast ILI trends in space and time (see Figure 5 in Multimedia Appendix 2).

Moving forward, it will be critical to define the threshold of localization for which Twitter can be a useful predictive dataset. For Twitter data to be validated, gold standard public health data must be made more available. Testing the correlative value for the flu cases at the NYC borough level begins to show the limits of Twitter’s capabilities in local ILI-ED prediction (see Figure 7 in Multimedia Appendix 2). With an expected increase in mobile devices and social media activity in the coming years, we look forward to the new challenges, insights, and applications gained from Twitter in the growing field of data-driven epidemiology.

## Multimedia Appendix 1

Examples of classified tweets.

## Multimedia Appendix 2

Search phrases for automated-searching; Consideration of weekday effects in temporal models; spatial models of sick tweeters within NYC and relation to vaccination sites; Temporal trends for Awareness-based data; Vector map construction for online Web application; and Gold standard, daily prediction at NYC borough level.

## Multimedia Appendix 3

Flu-related tweets and their associated codes.

## Abbreviations

A: Awareness tweet
AR: auto-regressive
GSQ: Google Trends search query
H: High probability of tweet veracity
I: Infection tweet
ILI-ED: influenza-like illness emergency department visit
L: Low probability of tweet veracity
M: Medium probability of tweet veracity
MAPE: mean absolute percent error
NYC: New York City
O: Subject of tweet is “other” person
R: Relevant tweet
S: Subject of tweet is self
