# Supplementary material for: A Case Study of the New York City 2012-2013 Influenza Season With Daily Geocoded Twitter Data From Temporal and Spatiotemporal Perspectives
Source: J Med Internet Res. 2014 Oct 20;16(10):e236. doi: 10.2196/jmir.3416 (PMC4259880; doi:10.2196/jmir.3416)
Supplement: Supplementary file 1 [file jmir_v16i10e236_app1.pdf]

Multimedia Appendix 1. Examples of classified tweets.

Label[a]	Example Tweets
Irrelevant	The flu shot prevents hangovers, so going all out w/5 fingers of...wait, it’s “no drinking” that prevents hangovers? #toolate
	Romney: I would not put no flu zones over Syria. Military is not necessary in the conflict #debate2012
RISH	This flu is kicking my butt, 2nd day off work. Hopefully I can win the battle because I’m losing sick days & that might hurt my pockets later
	Maldito flu sueltame!!! ðŸ˜«ðŸ˜¡
RISM	Uhhh I think I might be getting the flu /:
	Creo que me va a dar Gripe :'(
RISL	Finally getting over a miserae flu.
	@boyXsupreme ik it’s awful. the past two weeks darci and I have had the flu but thank god we’re done with it. get lots of rest + tlc ðŸ’•
RIOH	When @CiaraAnnex3 has the flu... Love you but stay the hell away <3
	Running on no sleep my poor daughter has the flu
RIOM	@giannarussso YOU PROB GOTS THE FLU!!, ariannas has it
	@YanieseRivera damn girl, do you have the flu?
RIOL	On day 6, son’s #flu is gone. He threw open side door and screamed to the outside, "FREEDOM"! Then shoveled snow. I am miserable on day 3.
	@_AlexAlford she's good too, fortunately she never actually got the flu. just a fever for a day or two
RASH	I’d rather get the flu than get the flu shot, #JustSayin. No needles for me.
	The flu is an epidemic here and I volunteer at a preschool twice a week. If I don’t get the flu it will be a miracle.
RASM	I survived more than a week in NYC without contracting the flu! Let’s hope the plane ride home won’t break me. Trying to stay healthy here.
	So glad to be back in NYC, but stay away from me you Flu filled city.

RASL	Ah yea... flu shot acquired! (@ Duane Reade) http://t.co/RSR2Bl11
	Just got a flu shot and Tdap booster at @onemedical â€” if youâ€™ll be in close contact with an infant, consider taking these vaccines.
RAOH	Flu infections sweep America hospitalizing thousands and leaving 18 children dead of complications, ... http://t.co/Yf9eQikm
	19,000 flu cases across NY this week. I'm not leaving my house.
RAOM	Wash your hand. America sick girl. #influenza #SICKENING #cleanup http://t.co/MKdASaV9
	The latest figures from the CDC show that flu cases are still rising in the west. Listen to our newscast: http://t.co/0kCiyoP8
RAOL	#patient advice. #flu vaccine not only protects you but your community Too. Less outbreaks. U may survive the flu, but sicker people may not
	â€œ@grubstreetny: Hereâ€™s How New York Chefs Beat the Flu http://t.co/bV3pEZ2Câ€ • uuuuu for real?

[a]Relevant (R), Awareness (A), Infection (I), Self (S), Other (O), High (H), Medium (M), Low (L).
