# Supplementary material for: A Case Study of the New York City 2012-2013 Influenza Season With Daily Geocoded Twitter Data From Temporal and Spatiotemporal Perspectives
Source: J Med Internet Res. 2014 Oct 20;16(10):e236. doi: 10.2196/jmir.3416 (PMC4259880; doi:10.2196/jmir.3416)
Supplement: Supplementary file 2 [file jmir_v16i10e236_app2.pdf]

Appendix

Table 1. Phrases for Natural Language Processing:

**Phrase**	**Category**
has the flu	RIOH/M
have the flu	RISH/M
having the flu	RISH/M
got the flu	RISH/M
get the flu	RASM
getting the flu	RASM
with the flu	RISH
feel sick	RISH/M
feeling sick	RISH/M
tengo gripe	RISH/M
tiene gripe	RIOH/M
tienes gripe	RIOH/M
this flu	RISH
flu has to go	RISH
public health emergency	RAOH
epidemic	RAOH
<expletive> flu	RISH
gave me the flu	RISH
flu shot	RASL/M
getting over the flu	RISM/L
tamiflu	RISH/M
gym	RISL
work out	RISL
news / .com	RAOM
cold/flu	RISM
i hope ... flu	RISM
i think ... flu	RISM

Weekday Effect

Weekday Effect was not strongly observed after factoring a week-to-week analysis. Each days z-score was calculated based on the relative number of infectious tweets on that day compared to the number of infectious tweets on other days in the week. The z-score was calculated for each day of the week, for 29 weeks. The following spaghetti plot shows qualitatively a lack of a strong central tendency across days of the week (with the mean in black).

Figure 6. Z-Scores of Each Day of the Week Calculated from Relative Frequency of Infection Based Tweets (Supplemental Figure 1)

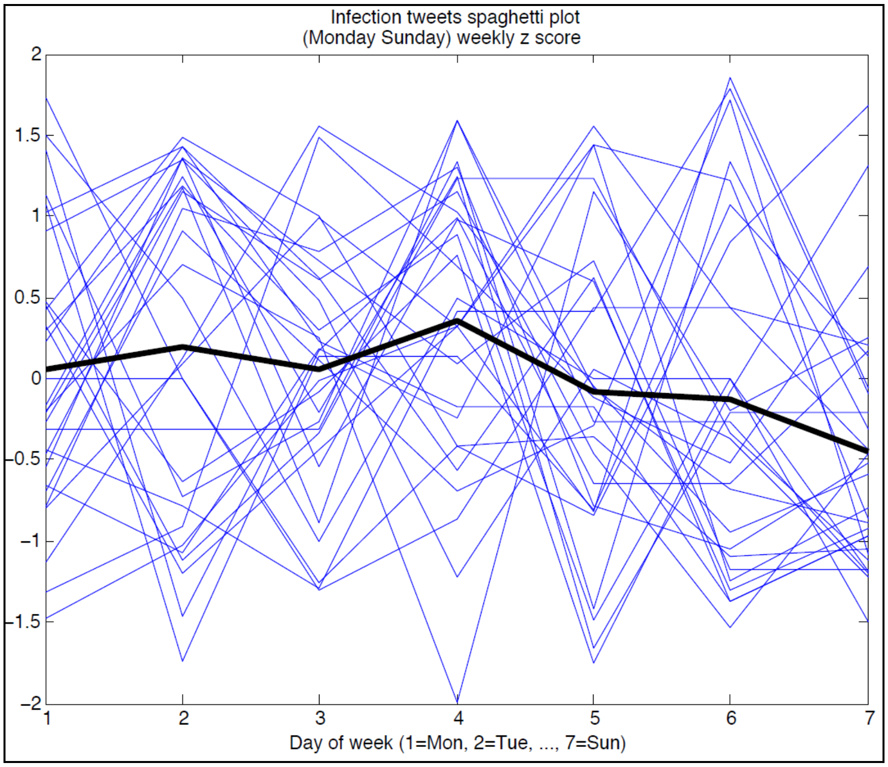

Figure 7. Relationships between ILI and Awareness Tweets (Supplemental Figure 2)

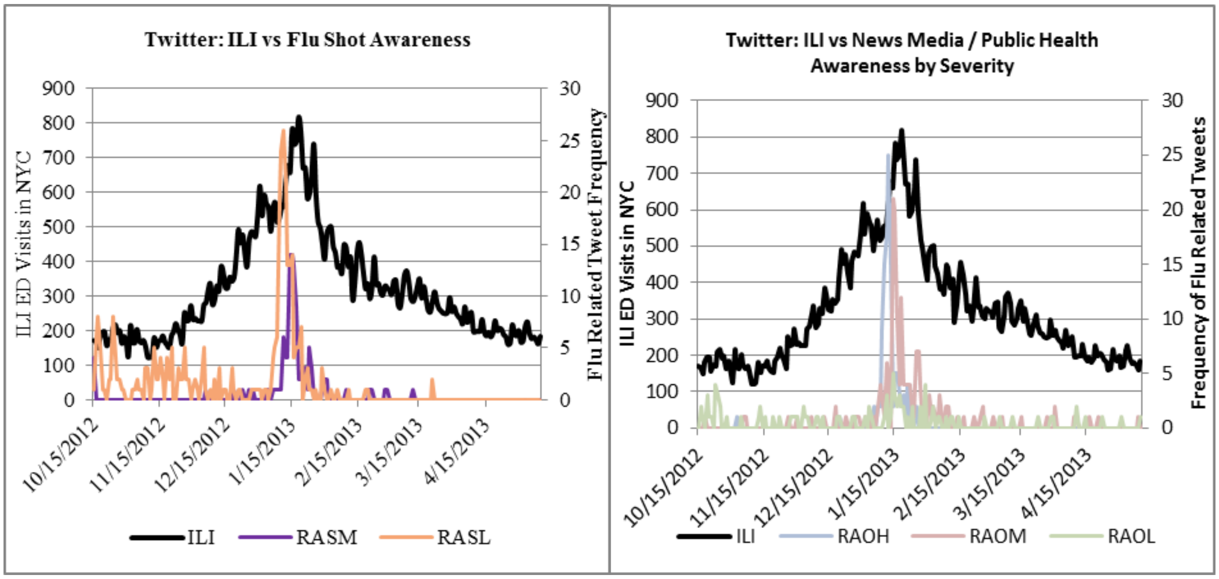

Many RASL tweets referred to immunization events. Note the prevalence in immunization event tweets (left) at the beginning of November, while the growth towards peak flu season has already been well underway.

Media related tweets can also show insights (right). High probability of sickness tweets in the form of ILI and other confirmed-case sickness reports were broadcast closer to peak flu season. This however does not leave much lead time for those anticipating which week that peek in ILI-ED visits will occur.

<u>**Spatial Models**</u>

Figure 8. Spatial Probability Prediction Map of RISH Tweets and Vaccine Site Availability (Supplementary Figure 3)

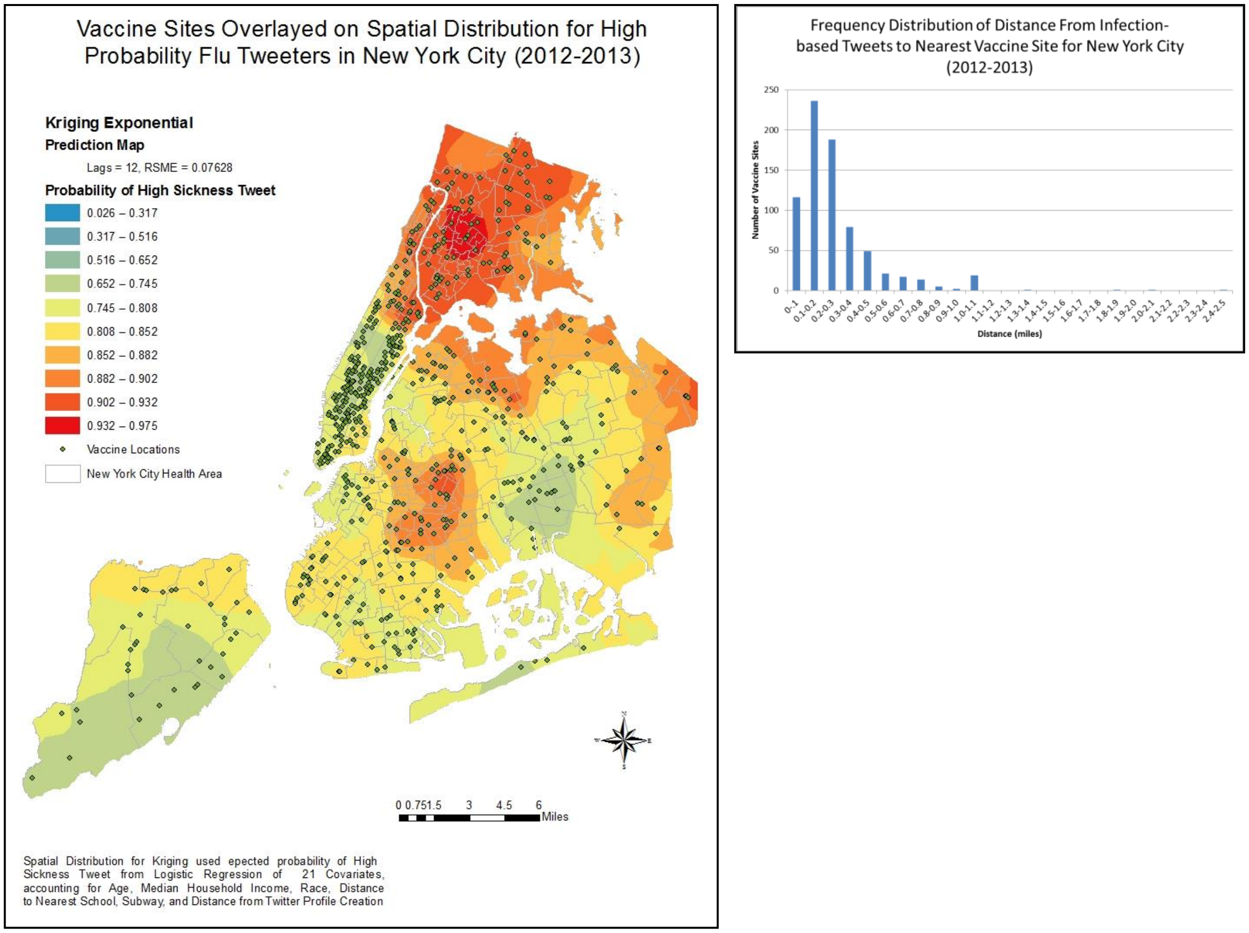

Methods

An exponential Kriging map was constructed using Arc-GIS software from 1185 RISH tweets with tweet-specific geolocation in New York City proper. This prediction map model was selected adjusted to minimize the RSME. Furthermore, 21 covariates were considered for the logistic regression to explain the spatial distribution. These variables included county level percentage data for age bracket, population density, median house hold income, race, and student absenteeism from enrollment. Additional variables included distance from nearest school and subway and distance to location of twitter profile creation (a proxy for distance from home location). The rationale was to consider socioeconomic and ethnic factors that could predispose infection and thereby predispose infection related tweets. Distances from nearest school and subway stop were considered because these locations present areas of mass gathering and

therefore potential hotspots of infectious disease transmission. Distance from twitter profile location was considered because those who are sicker could potentially be more likely to tweet from home. Covariate data was obtained from Bytes of the Big Apple and SimplyMap. Finally, a distance raster was also created to find the distribution of nearest influenza vaccine sites from each RISH tweet. Vaccine sites were obtained from NYC DOH's FluLocator tool and geocoded with Mapquest.

Results

The spatial distribution alone, after adjusting for covariates, suggested that higher concentrations of high probability infection tweeters could be found in Harlem and Southwest Bronx (see Figure 4 below). The prediction error for most of NYC was low (P = 0.03-0.06). Percent African American had the highest risk ratio for prediction probability of Infection-tweet spatially, but none of the covariates were found to be statistically significant (confidence intervals included 1 for relative risk ratio). The distribution of vaccine distances was skewed towards higher values. It is not clear if the distribution is Poisson given few vaccine sites within 0.1 miles of the tweets. The median distance from the vaccine sites to RISH tweets was 0.22 miles, but the density of vaccine sites was not high in spatially predicted hotzones (probability of a RISH tweet 0.882+) compared to Lower Manhattan (probability from 0.652-0.882).

Discussion

Spatial analysis of geocoded tweets suggests areas of high probability of RISH tweets down to the street level. This could provide a starting point for areas to target for stronger vaccine coverage. Discrepancy between vaccine store availability and probability of high sickness tweet in the same locality is apparent. This may be explained more by a function tweeter demographics and tweeter behavior than a function of distance to vaccination sites where the median was only 0.22 miles. Of the covariates explored to explain the RISH-tweet prediction map, socioeconomic, demographic, and distance from mas gatherings of the tweeters all failed to have any association. This could suggest the high level of transmissibility of the disease. Other covariates, not considered here, such as weather patterns and route of commute may also be predictors.

One clear limitation of this model is the assumption that disease (and by proxy, sick tweeters) are distributed continuously. It is not clear how patterns of commute and other dynamics of transmission affect how spread of flu operates on a macro scale. Individual-based models have been proposed by Sadilek and colleagues to account for colocation with other sick tweeters and sick friends within one's network. These models also have yet to be validated for the macro-level effects due to lack of gold standard data. If we consider sickness tweets as static events rather than foot-prints, a kernel density map may provide a better representation of the distribution of sick tweeters. Below in Figure 9, one can observe the overlap between vaccine distribution sites and the locations of high-probability sick tweeters. Areas in Bronx, Southwest Harlem, and South East Queens in particular show high levels of sick tweeters without corresponding high

levels of vaccine sites. On the other hand, Midtown, West Manhattan, Northern Brooklyn show dense clusters of vaccine sites. It is unclear if there is a significant association given the mean distribution to the nearest vaccine site is 0.22 miles (as shown in Figure 8 above).

Figure 9. Kernel Density Estimate of High Probability Sick Tweeters (Supplementary Figure 4)

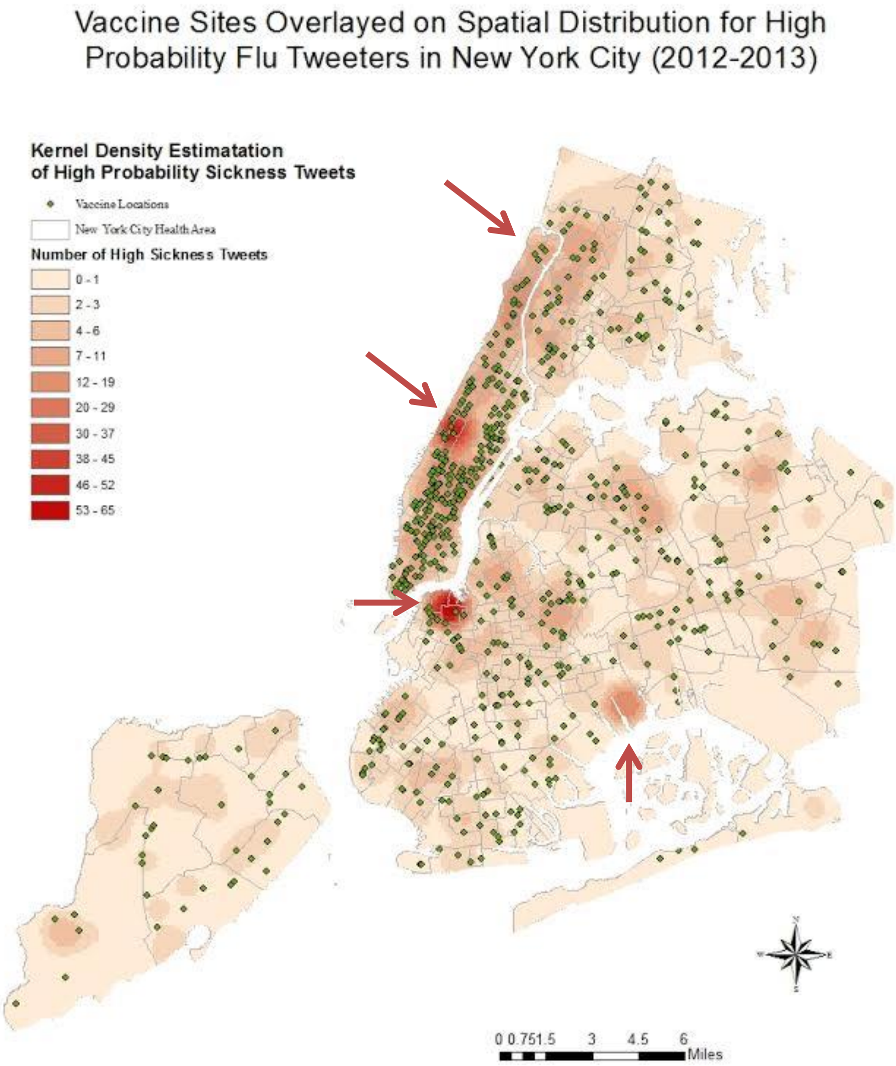

Figure 10. Vector-map and Population-normalized Heatmap for Spread of Influenza Related Tweets (Supplemental Figure 5)

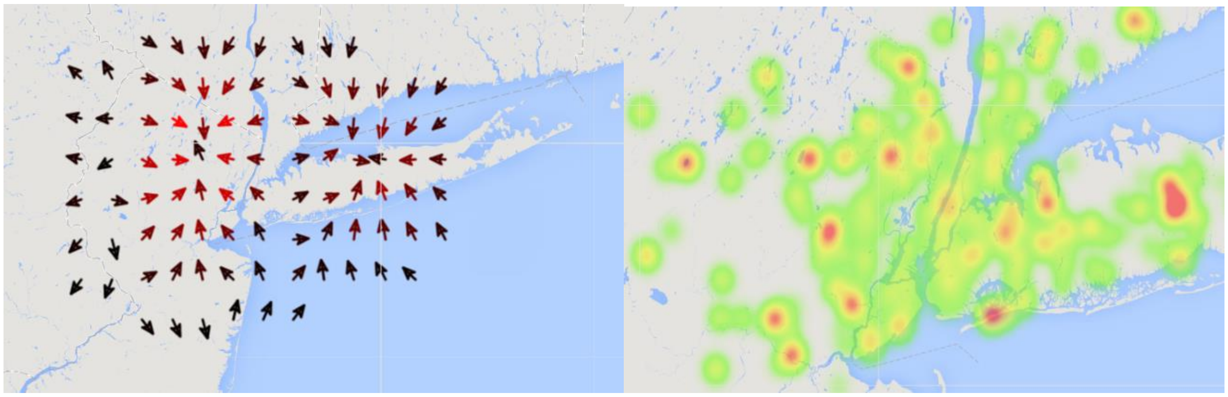

The images above show the results of two models being developed to visualize the spread of flu-related tweets on an web-based application in beta-testing to be integrated into the HealthMap.org framework. The image above represents a snapshot of the week of 1/8-1/15 (right) and 1/2 -1/9/13 (left). A slider (not shown) can be used to track movement dynamically from week to week. Vectors point towards direction of greatest positive percent change in tweet frequency in the neighboring grids and are colored more red for the proportional percent change.

The HeatMap implements the JavaScript HeatMap API for Google Maps. With incorporation of the slider, the HeatMap allows for dynamic forecasting, by showing how disease hot spots change with respect to time. For New York City, population data has also been recorded for 0.01 degree grid boxes, allowing normalization of the HeatMap against population data. Vector-map functions by calculating the percent change in each 0.01 degree area from the previous 7 day period. For each area the percent change is compared to the percent change in surrounding grid boxes. A vector is constructed to point towards areas of higher, positive change and away from areas of lower, negative change. The stroke color of each vector is relative to that area's percentage change. The rationale behind the Vector-map was to display the movement of tweets directly. The principal assumption behind the Vector-map design is that the local equilibria are maintained in that the decrease of tweets in low areas is a result of tweets and by extension disease, moving towards areas of higher activity.

Figure 11. ILI Emergency Department Visits by Borough from New York Department of Health and Hygeine (Supplemental Figure 6)

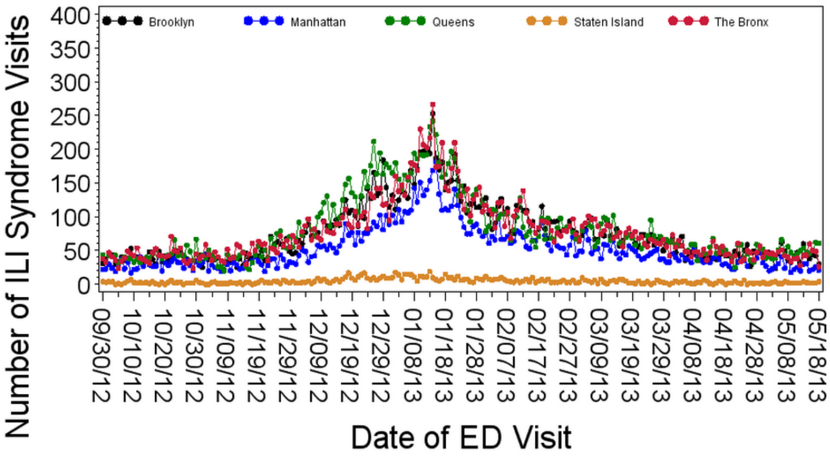

Figure 12. Twitter Prediction at the Borough Level (Supplemental Figure 7)

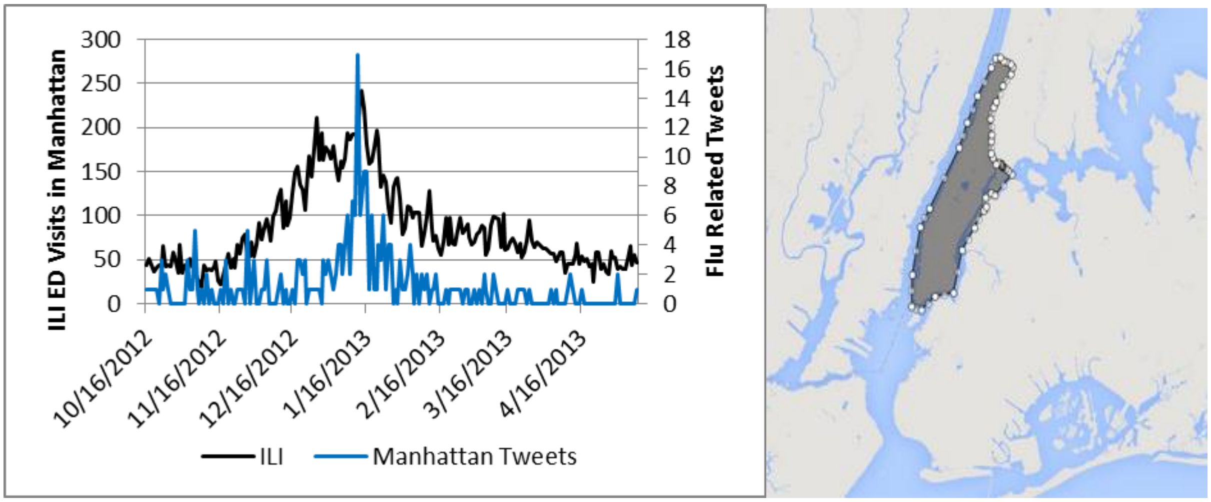

A time series was extracted by the area inside the shaded polygon drawn above. Pearson correlation between 10/15/2012 and 5/18/2013 for Manhattan tweets and Manhattan ILI-ED visits was 0.677 and 0.55 for Queens. These two boroughs had the most data (Relevant tweets) for location based queries for influenza indicators as used for the greater NYC region. Neither time series data set was stable by ADF. An AR model for Manhattan was able to produce a MAPE of 14.5 for the training set of 10-15 to 02-04 and a prediction set of 02-05 to 02-07-2013. This example demonstrates the limits of Twitter's predictive abilities as the area of evaluation is increasingly localized. It is therefore important to define thresholds for what constitutes real signal. To our knowledge, this is the most localized use case of Twitter for with some predictive ability for ILI-ED visits.
